# Water Quality Criteria and Ecological Risk Assessment of Typical Transition Metals in South Asia

**DOI:** 10.3390/ijerph192316125

**Published:** 2022-12-02

**Authors:** Ying Wang, Tanjena Rume, S. M. Didar-Ul Islam, Wenhong Fan, Jiangyue Wu, Xiaomin Li

**Affiliations:** 1 School of Space and Environment, Beihang University, Beijing 100191, China; 2 School of Civil Engineering and Geosciences, Newcastle University, Newcastle upon Tyne NE1 7RU, UK; 3 Beijing Advanced Innovation Center for Big Data-Based Precision Medicine, Beihang University, Beijing 100191, China; 4 National Marine Hazard Mitigation Service, Ministry of Natural Resource of the People’s Republic of China, Beijing 100194, China

**Keywords:** South Asia, metals, acute and chronic water quality criteria, NPKDE-SSD, water quality standard, ecological risk, transboundary river

## Abstract

Transition metal pollution in rivers in South Asia is more serious than in other regions because of the lack of adequate freshwater management measures. Water quality criteria (WQC) for South Asia is urgently needed to protect regional aquatic environments because of the occurrence of transboundary rivers. The present study established non-parametric kernel density estimation species sensitivity distribution (NPKDE-SSD) models and then derived the acceptable hazardous concentration for protection of 95% of all aquatic species (HC5) and WQC of six typical transition metals in South Asia. The results showed that the order of acute and chronic WQC was Mn > Fe > Cd > Zn > Cu > Hg and Cu > Fe > Cd, respectively. A risk assessment of these metals in the Indus River, the Ganges River, the Brahmaputra River, the Meghna River, and the Bagmati River was also carried out. Based on the results, these major rivers in South Asia were highly polluted with transition metals, with significant ecological risks for a large number of aquatic species. This study can contribute to a better understanding of ecological risks in South Asia and provide a scientific basis for the updating of water quality standards and the increase in overall water quality.

## 1. Introduction

Transition metals are widespread in aquatic environments and can adversely affect aquatic organisms and human health when their concentrations exceed threshold values [1,2,3]. Water pollution caused by transition metals, which generally originate from industrial discharge and mining activities, has recently attracted considerable global attention [4]. In South Asia, e.g., Bangladesh, India, Pakistan, and Nepal, because of the lack of adequate sewage treatment technologies and the excessive industrial production of all sorts of goods, waterbodies are frequently polluted with transition metals [5]. For instance, the peripheral rivers of Dhaka, the capital of Bangladesh, are highly contaminated with iron (Fe), manganese (Mn), nickel (Ni), zinc (Zn), copper (Cu), and lead (Pb) [6,7,8], with harmful impacts on the natural environment. It also happens in the water environment of India, Pakistan, and Nepal [9,10,11]. 

The water quality criteria (WQC) are the maximum acceptable threshold values recommended for chemical substances or environmental parameters for protecting specific water bodies functions and organisms from adverse effects [12]. Several countries, including the United States, Canada, Australia, and China, have established their own WQC with double or single threshold value systems. Double threshold values include acute WQC and chronic WQC, often using criteria maximum concentrations (CMCs) and criteria continuous concentrations (CCCs) as thresholds for determining acute and chronic injury, respectively, for aquatic organisms [13]. However, countries such as Bangladesh, India, Pakistan, and Nepal have not established WQC, mostly because of the occurrence of transboundary rivers. The major south and southeast Asian river basins (Indus, Brahmaputra, and Ganges) arise from the Himalayas and benefit over 1 billion people [14]. These rivers connect multiple countries, e.g., the Indus basin of India, Pakistan, and Afghanistan. The Brahmaputra and Ganges basins connect Bangladesh, Bhutan, India, and Nepal. In recent decades, shared river basins have sparked debates among South Asian countries in terms of the transboundary movement of toxic pollutants. In this sense, WQC for South Asia, but not a country in South Asia, are urgently needed to protect and assess the ecological risks for aquatic environments.

Species sensitivity distribution (SSD) is a statistical approach that is widely used to derive WQC and assess specific risks. It is estimated from a sample of toxicity data for various species that are used as surrogates and assumed to represent the community structure of a specific ecosystem and visualized as a cumulative distribution function (CDF) to describe the SSD curve [15]. SSDs were widely applied to derive WQC for several transition metals, such as Cu, Ni, Zn, and cadmium (Cd), to protect freshwater aquatic organisms, but rarely used to derive WQC for multiple pollutants [2,3,12]. The predicted no-effect concentration (PNEC) can also be calculated via the SSD and the entropy method for ecological risk assessment [4]. In our previous study, we proved that a non-parametric kernel density estimation (NPKDE)-SSD better reflects the overall trend of changes in species toxicity data than parametric models and is more adequate in determining the internal structural characteristics of toxicity data [16]. For sensitive species, an NPKDE-SSD also yields better fits, more robustness, and better predictions than parametric models, making the calculated WQC more reliable [3,4].

Hence, the present study aims to determine the WQC and assess the ecological risks of six typical transition metals, i.e., Cd, Cu, mercury (Hg), Mn, Fe, and Zn, in South Asia. Their acute and chronic WQC for better water management were derived by the use of NPKDE-SSD models. Based on these threshold values, the ecological risks of these typical transition metals in the major South Asian rivers were assessed to investigate the transition metal pollution status and environmental risk. 

## 2. Materials and Methods

### 2.1. Datasets

For the present study, toxicity data of the selected transition metals to aquatic organisms in South Asia and water quality data of the major South Asian rivers were collected. Acute and chronic toxicity data of the selected transition metals of freshwater aquatic organisms were obtained from the USEPA ECOTOX database (http://cfpub.epa.gov/ecotox/) up to the 13 July 2020. The data accuracy and reliability were evaluated using standard methods [17], based on WQC guidelines and literature [18]. The selection of toxicity data was based on the following principles: (1) selected species contains South Asian indigenous species, introduced species, and internationally common/standard test species; (2) selected species covers at least three trophic levels (i.e., aquatic plants/primary producer, non-vertebrate/primary consumer, vertebrate/secondary consumer); (3) the lethal concentration affecting 50% of individuals (LC_50_) or the effective concentration affecting 50% of individuals (EC_50_) were selected as the acute toxicity endpoint, whereas the no observed effect concentration (NOEC) or the lowest observed effect concentration (LOEC) were selected as the chronic toxicity endpoint; and (4) toxicity data should be semi-static or static experimental data. The geometric mean was calculated as species mean acute/chronic values (SMAV/SMCV) if there were multiple toxicity datasets for the same species [19]. The measured concentrations of different heavy metals in the typical South Asian rivers (i.e., the Indus, the Ganges, the Brahmaputra, the Meghna, and the Bagmati River) with the confluence of different countries (i.e., Bangladesh, India, Pakistan, and Nepal) were also collected based on previously published results.

### 2.2. SSD Modeling

Different species show differences in sensitivities to the same chemical constituents, and this variation can be defined as SSD. In this study, NPKDE-SSDs were constructed based on our previous study [3], in which NPKED-SSDs have been proven could better reflect the overall trend of changes in species toxicity data than parametric models. The goodness-of-fit of SSDs were evaluated with the Kolmogorov–Smirnov (K-S) test, root mean square error (RMSE), and error sum of squares (SSE). The model was considered sufficient (i.e., the null hypothesis is not rejected, that the empirical data fit the hypothetical cumulative distribution) when the *p*-value for K-S test (*P_KS_*) was greater than 0.05. SSDs modeling processes and goodness-of-fit tests were carried out by the use of MATLAB R2019b (Mathworks, Natick, MA, USA).

### 2.3. HC5 and WQC Derivation

If the proportion of aquatic species protected is set at 95%, the acceptable concentration of the contaminant is hazardous for the remaining 5% and is therefore defined as HC5 [2,3,20], which is the concentration value corresponding to 5% cumulative probability on the SSD curve. Acute and chronic HC5 values are the basis for deriving acute and chronic WQC [20], which were calculated using an assessment factor of 2 to avoid uncertainties (Equation (1)) [21]:

WQC = HC5/2(1)

### 2.4. Ecological Risk Assessment

The SSD method can also be used to conduct environmental risk assessments by the use of the potentially affected fraction (PAF) value, which is the cumulative probability calculated based on the established SSDs [22]. The PAF in the present study was calculated based on NPKDE-SSD using Equation (2).

(2) $$\mathrm{PAF}\approx \frac{1}{1.06\sigma_{0}n^{4/5}}\sum_{i=1}^{n}\frac{1}{\sqrt{2\pi }}e^{-\frac{{\left(\frac{C-x_{i}}{1.06\sigma_{0}n^{-1/5}}\right)}^{2}}{2}}$$

where *C* is the log-transformed exposure concentration, *n* is the sample size, *σ*_0_ is the standard deviation of the log-transformed toxicity data, and *x_i_* is the *i*th sample of log-transformed toxicity data.

The ecological risk is high when the PAF value is greater than or equal to 0.05; otherwise, it is low or insignificant.

## 3. Results and Discussion

### 3.1. Development of Acute and Chronic SSDs of Typical Transition Metals

Overall, there were 281 species with acute toxicity data for Cd (76), Cu (66), Hg (59), Mn (12), Fe (13), and Zn (55), including plants (22), fish (114), mollusks (32), amphibians (23), crustaceans (46), insects (12), invertebrates (20), and worms (10) (Table S1). Chronic toxicity data were available to a much lower extent. We only collected a total of 88 species with chronic toxicity data for Cd (34), Cu (38), and Fe (16) (Table S2), containing plants (18), fish (30), mollusks (9), crustaceans (13), insects (7), invertebrates (4), worms (5), and amphibians (2) (Table S2). It showed that more data were available for temperate than for tropical species. Since the ranges were relatively large, the acute and chronic toxicity data were log_10_-transformed to reduce the relative difference for these metals, facilitating calculations.

**Figure 1:**
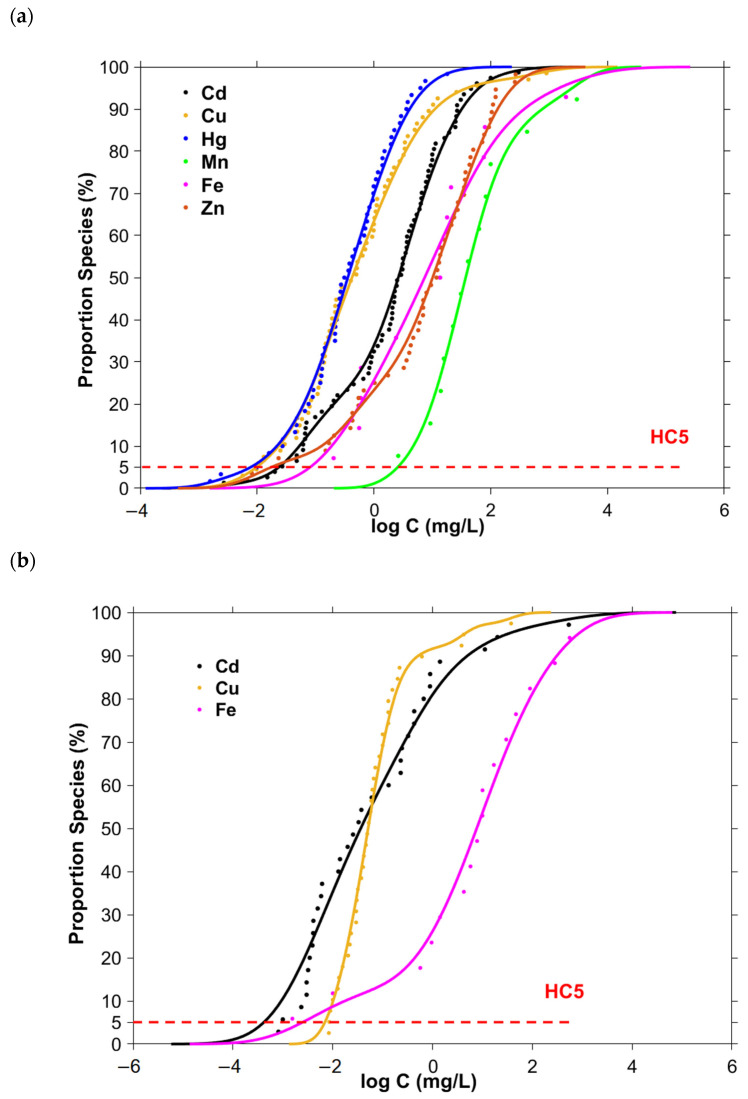
NPKDE-SSDs for typical transition metals in South Asia. (**a**) Acute NPKDE-SSDs for six typical transition metals (Cd (●), Cu (●), Hg (●), Mn (●), Fe (●), and Zn (●)) in South Asia. (**b**) Chronic NPKDE-SSDs for three typical transition metals (Cd (●), Cu (●), and Fe (●)) in South Asia.

The acute and chronic NPKDE-SSDs of six and three typical transition metals, respectively, in South Asia were established (Figure 1). The *P*_ks_ values of the NPKED-SSD models were all greater than 0.05, and the RMSE and SSE values were minimal (Table 1), indicating that the NPKED-SSD models developed for these metals showed a good fit, high accuracy, and suitability for use in hazard simulation.

### 3.2. Derivation of Acute and Chronic HC5 Values for South Asia

The acute and chronic HC5 values were derived based on SSDs to establish the acute and chronic WQC of aquatic organisms for the studied metals (Table 1). It is found that the order of the acute HC5 values is Mn (2.56 mg/L) > Fe (81.1 μg/L) > Cd (23.6 μg/L) > Zn (17.5 μg/L) > Cu (9.8 μg/L) > Hg (7.8 μg/L) (Figure S1a), indicating that Hg has the strongest acute toxic potency among these six transition metals to aquatic organisms in South Asia. As a global pollutant, Hg is persistent, highly mobile through air transport, and can bioaccumulate in organisms and food chains [23]. Although the mechanism of Hg toxicity is still largely unclear, it might be related to the fact that Hg binds to thiol groups in proteins and thus affects the enzymatic activities of organisms [18]. In addition, the toxic potencies of Fe and Mn to aquatic organisms in South Asia were low, which is consistent with our previous results on freshwater fish [3]. However, the acute HC5 values differed from those obtained in our previous study, along with a different order (i.e., Mn > Zn > Cu > Fe > Cd > Hg) [2,16]. This can be explained by the differences in regional species and the available data. The sensitive species near 5% cumulative probability for Cd, Cu, and Hg are crustaceans, and for Mn, Fe, and Zn are plants, worms, and fish, respectively. Because of the warm climate in South Asia, metals may show different toxicity levels to tropical species. Only the HC5 values of Cu and Zn were lower than those reported in previous studies, whereas the HC5 values of the other four metals were higher. Some studies have shown that in freshwater environments, temperate species are more sensitive to most metals than tropical species, although tropical species are more sensitive to Zn [24,25]. According to a previous study, the toxic potencies of Cd, Cu, Zn, and Hg to marine species are directly proportional to the temperature [26]. This explains the need to establish regional WQC [12]. 

The chronic HC5 values followed the order Cu (6.9 μg/L) > Fe (2.4 μg/L) > Cd (0.413 μg/L) (Figure S1b), similar to the acute HC5 values reported in our previous research [2,16]. Of these, Cd showed the strongest chronic toxic potency to aquatic organisms in South Asia. In aquatic organisms, long-term exposure to Cd can lead to adverse effects on growth, reproduction, immune and endocrine systems, and behavior [27,28]. In addition, Fe showed a stronger toxicity than Cu, which can be explained as follows: (1) due to the lack of data, we used more model biological toxicity data in the construction of chronic SSDs. Therefore, the sensitive species near 5% cumulative probability for Fe is *Oncorhynchus mykiss* (1.5 μg/L), which is globally distributed, but not common in South Asia; (2) the dataset used for Fe was small, resulting in a deviation of building SSD. However, further studies comparing the chronic toxicities of Fe and Cu to temperate and tropical freshwater species are necessary since we could not adequately protect tropical species by thresholds derived from temperate species [29].

### 3.3. Derivation and Comparison of WQC

Acute and chronic WQC were derived based on the HC5 and compared with those found in other publications (Table 2). The acute WQC values of Cd and Hg were greater than the threshold values recommended by the USEPA [30], whereas the acute WQC values of Zn and the chronic WQC values of Cd and Fe were below the threshold levels. In addition, the WQC derived in this study were also significantly differed from the threshold values published previously, indicating differences among South Asian WQC and those of other countries. These differences may be due to three reasons. First, it can be explained by the differences in the datasets and the species used to establish the SSDs, indicating that species compositions and their relative sensitivities largely contribute to the building of the SSD. Second, the toxicity of pollutants can be affected by various environmental factors [31,32], such as the hardness of the water [33], which was not considered in the present study as the data were inefficient. Third, the non-parametric, kernel density estimation might be not very appropriate to use with a small sample size (<30) [16], such as for Mn and Fe. However, the results still indicated that there is an urgent demand for distinguished regional WQC for South Asia to assess the ecological risks of water environments.

### 3.4. Ecological Risk Assessment of Transition Metals in Typical South Asian Rivers

The major southern and southeastern Asian river basins (i.e., Indus, Brahmaputra, and Ganges) arise from the Himalayas and benefit over 1 billion people [14]. These rivers are transboundary rivers, such as the Brahmaputra and Ganges basins, which connect Bangladesh, Bhutan, India, and Nepal. South Asian rivers including these transboundary rivers are highly polluted with various metals via the input of domestic, agricultural, and industrial wastes [10,39,40,41]. For example, the Pb concentration detected in the Karnaphuli River in Bangladesh ranges from 5.29–27.45 µg/L in water and 21.98–73.42 mg/kg in sediments [41]. However, the transboundary movement of toxic pollutants from such rivers is a matter of concern. In recent decades, the transformation of contaminants between upstream and downstream countries has sparked debates among South Asian countries [14]. The contamination of rivers with metals may have devastating effects on the ecological balance of the aquatic environment, decreasing the diversity of aquatic organisms [8,42]. In this sense, the use of unified toxicity data and WQC to assess the ecological risk of transboundary rivers may be the first step to solving this issue. However, in South Asia, there is not only no available information on WQC, but also, some countries do not even have surface water quality standards (WQSs). The governments of these countries only established national drinking WQSs to protect human health [43,44,45,46,47]. In order to investigate the transition metals pollution status in major South Asian rivers, in this study, water quality data of the major South Asian rivers (i.e., the Indus, the Ganges, the Brahmaputra, the Meghna, and the Bagmati River) with the confluence of different countries (i.e., Bangladesh, India, Pakistan, and Nepal) for the six studied metals were collected from recent publications and official reports or websites (Table 3). Then, the ecological risks of typical transition metals in these major South Asian rivers were evaluated based on NPKDE-SSDs established in the present study.

**Figure 2:**
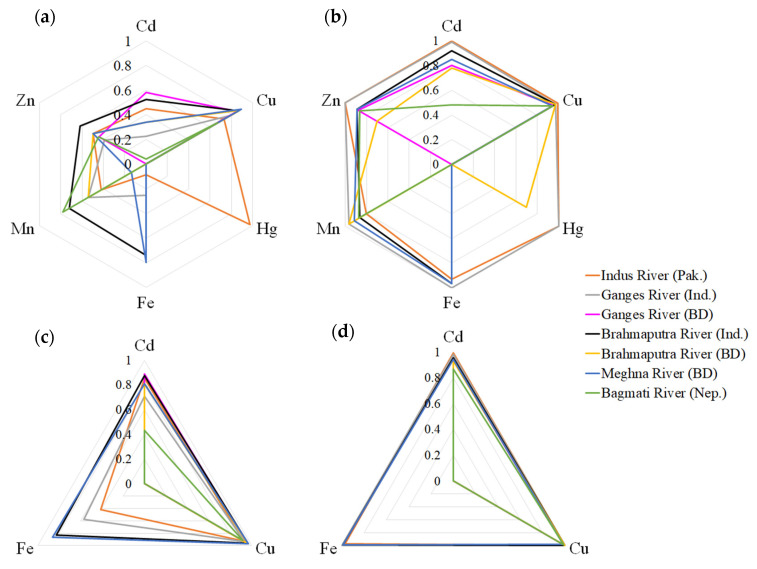
Spider diagram showing the trends of acute and chronic ecological risks of transition metals in different rivers (Indus River (Pakistan) (-), Ganges River (India) (-), Ganges River (Bangladesh) (-), Brahmaputra River (India) (-), Brahmaputra River (Bangladesh) (-), Meghna River (Bangladesh) (-), Bagmati River (Nepal) (-)) in South Asia. (**a**) Lowest acute PAF values of six metals in different rivers; (**b**) highest acute PAF values of six metals in different rivers; (**c**) lowest chronic PAF values of three metals in different rivers; (**d**) highest chronic PAF values of three metals in different rivers.

The collected data for Cd, Cu, and Zn were abundant and complete, which was not the case for the Hg data. It is disconcerting that the sample size of Hg is so small, since Hg is recognized as a global pollutant. We speculated that it is possibly related to the higher complexity for determination than the other metals. The Ganges River in India was sampled most commonly, where the same river in Bangladesh had the lowest sample size. According to the ecological risk assessment via the use of the constructed acute and chronic NPKDE-SSD models of typical transition metals, the acute PAF values of all metals in almost all rivers were above 0.05, and the PAF values of the maximum detected concentrations exceeded 0.69 (Figure 2). The ecological risks of long-term exposure to Cd, Fe, and Cu are extremely high; particularly for Cu, the PAFs of the detected minimum concentrations all exceeded 0.9 (Figure 2c,d), resulting in significant threats to aquatic organisms. When comparing the average concentrations of the six transition metals detected in each river with the standards of their respective countries [43,44,45,46], only the Bagmati River (Nepal) did not exceed the standard for all metals (except for Hg and Fe due to the lack of available data). Due to the high standard value of Zn, none of the rivers exceeded the threshold. However, most of the remaining metals detected in the rivers exceeded the standard, and the rivers Ganges and Brahmaputra, passing through India and Bangladesh, showed the highest levels.

The Ganges River, named Ganga in India and Padma in Bangladesh, originates from the Gangotri glacier at Gomukh in the province Uttarakhand of India [75]. The water of the Ganges River in India is subject to substantial pollution through the input of untreated domestic and industrial wastes [76,77]; the concentrations of Fe, Mn, Hg, and Cd are high. The concentrations of Cd, Hg, and Fe detected in the Ganges River in India were the highest, with average values of 0.029, 0.14, and 7.74 mg/L, respectively, exceeding the national WQSs of India by more than five times [43]. The water quality of the Ganges (Padma) River in Bangladesh is better than that in India (Figure 2). The Brahmaputra River is a large trans-Himalayan river, originating from Tibet and running through parts of China, Bhutan, India, and Bangladesh before flowing into the Bay of Bengal [67,68]. The Brahmaputra River in India is contaminated with Fe, Mn, Cu, Pb, Cd, Cr, As, and Ni, mainly via untreated wastewater, sewage, and effluents from municipalities and industries from the nearby catchment areas [48,49,50,67]. The risk of Mn pollution is highest in the Brahmaputra River of Bangladesh, with average Mn concentrations 20 times higher than those in upstream India. Previous studies have also shown that Mn is a common natural contaminant of groundwater in Bangladesh, where the maximum Mn level was 4.11 mg/L (mean, 0.53 mg/L) [78], which partly explains the high infant mortality rate in Bangladesh [79]. It also demonstrated that the current WQSs and other freshwater management measures can not protect aquatic organisms, and also human health, which should be updated and new tools adopted in time.

## 4. Conclusions

The present study is the first to obtain the acute WQC of six transition metals, i.e., Cd, Cu, Hg, Mn, Fe, and Zn, and the chronic WQC of three transition metals, i.e., Cd, Cu and Fe, in South Asia via the NPKDE-SSD method. Both acute and chronic risk assessments of transition metals in the major rivers in South Asia were performed, showing that in these rivers, transition metals pose a high ecological risk to aquatic organisms, endangering large numbers of species. In addition to the national drinking WQSs, it is also necessary to establish water quality standards based on WQC to protect aquatic organisms in the selected countries. Although the results remain open to experimentation and applications in the future, the present study still helps to better understand the ecological risks of metals in South Asia and provides a scientific basis for the protection of aquatic organisms and water environmental management in this region.

## Figures and Tables

**Table 1:** Overview of the acute and chronic NPKDE-SSDs of transition metals in South Asia. Parameters including the distribution parameter (bandwidth), goodness-of-fit evaluation results of the model (*p*-value of Kolmogorov–Smirnov (K-S) test, root mean square error (RMSE), and error sum of squares (SSE)), and HC5 values.

Classification	Metals	Bandwidth	*P_K-S_*	RMSE	SSE	HC5 (μg/L)
Acute	Cd	0.3522	0.896	0.0221	0.0371	23.6
	Cu	0.4057	0.934	0.0228	0.0344	9.8
	Hg	0.3689	0.965	0.0204	0.0246	7.8
	Mn	0.3679	0.985	0.0278	0.0093	2559.9
	Fe	0.7088	0.955	0.0501	0.0327	81.1
	Zn	0.3981	0.925	0.0213	0.0249	17.5
Chronic	Cd	0.7166	0.614	0.0434	0.064	0.41
	Cu	0.2664	0.981	0.0235	0.021	6.9
	Fe	0.6860	0.981	0.0336	0.018	2.4

**Table 2:** Comparison of acute and chronic water quality criteria of transition metals of South Asia with values found in other studies.

	Metals	Deriving Method	WQC (μg/L)	Literature
Acute	Cd	**NPKDE-SSD**	**11.8**	**This study**
		Slogistic3	0.42	[34]
		Burr ΙΙΙ-SSD	2.26	[35]
		Percentage of toxicity sorting	1.8	[30]
		Normal-SSD	3.8	[16]
		Logistic-SSD	5.23	
		NPKDE-SSD	1.7	
	Cu	**NPKDE-SSD**	**4.9**	**This study**
		BurrIII-SSD	2.25	[35]
		Normal-SSD	7.74	[2]
		Logistic-SSD	8.11	
		NPKDE-SSD	10.21	
	Hg	**NPKDE-SSD**	**3.9**	**This study**
		Log-slogistics-SSD	1.74	[18]
		Burr III-SSD	3.33	[35]
		RIVM-SSD	0.42	[36]
		Percentage of toxicity sorting	1.4	[30]
		Normal-SSD	1.73	[16]
		Logistic-SSD	1.91	
		Log-normal-SSD	1.99	
		Sigmoid-SSD	1.86	
		NPKDE-SSD	1.07	
	Fe	**NPKDE-SSD**	**40.6**	**This study**
		Normal-SSD	12.04	[2]
		Logistic-SSD	74.91	
		NPKDE-SSD	7.5	
	Mn	**NPKDE-SSD**	**1280**	**This study**
		Normal-SSD	100.76	[2]
		Logistic-SSD	100.2	
		NPKDE-SSD	76.03	
	Zn	**NPKDE-SSD**	**8.75**	**This study**
		Normal-SSD	36.16	[2]
		Logistic-SSD	50.97	
		NPKDE-SSD	17.935	
		Evaluation factors	30	[37]
		Percentage of toxicity sorting	120	[30]
Chronic	Cd	**NPKDE-SSD**	**0.207**	**This study**
		Percentage of toxicity sorting	0.72	[30]
		SSD	0.21–0.23	[34]
	Cu	**NPKDE-SSD**	**3.5**	**This study**
		SSD	9.44	[38]
	Fe	**NPKDE-SSD**	**1.2**	**This study**
		Percentage of toxicity sorting	1000	[30]

Note: The bold were the results obtained in the present study.

**Table 3:** Summary of the detected concentrations (unit: mg/L) collected from previous studies and acute and chronic PAF values calculated based on established NPKDE-SSDs of six transition metals in five South Asian rivers.

Metals	Statistics	Indus River (Pakistan)	Ganges River (India)	Ganges River (Bangladesh)	Brahmaputra River (India)	Brahmaputra River (Bangladesh)	Meghna River (Bangladesh)	Bagmati River (Nepal)	References
Cd	N ^a^	88	227	4	82	15	19	34	[40,48,49,50,51,52,53,54,55,56,57,58,59,60,61,62,63,64,65,66]
	Range ^b^	0.002–1.33	0.0003–0.33	0.004–0.014	0.003–0.041	0.001–0.012	0.001–0.02	0.00002–0.0024	
	GM ^c^	0.013	0.029	0.008	0.0049	0.0055	0.01	0.00038	
	SD ^d^	0.19	0.032	0.004	0.003	0.005	0.008	0.62	
	Acute PAF ^e^	0.79	0.89	0.72	0.62	0.65	0.75	0.24	This study
	Chronic PAF ^f^	0.93	0.95	0.92	0.90	0.90	0.92	0.73	
Cu	N ^a^	88	239	4	82	15	11	34	[40,48,49,50,51,52,53,54,55,56,57,58,59,61,62,63,64,67,68,69]
	Range ^b^	0.002–1.21	0.003–0.17	0.006–0.045	0.007–0.21	0.008–0.2	0.01–0.03	0.0013–0.037	
	GM ^c^	0.25	0.033	0.019	0.062	0.065	0.024	0.012	
	SD ^d^	0.435	0.026	0.01	0.035	0.055	0.011	9.1	
	Acute PAF ^e^	0.97	0.95	0.93	0.96	0.96	0.94	0.90	This study
	Chronic PAF ^f^	1.00	0.98	0.98	0.99	1.00	0.98	0.97	
Hg	N ^a^	14	30	/	/	12	/	/	[40,49,52,54,57,58,64,67,70]
	Range ^b^	0.014–2.35	0.0–0.49	/	/	0–0.001	/	/	
	GM ^c^	0.12	0.14	/	/	0.001	/	/	
	SD ^d^	0.082	0.076	/	/	0.001	/	/	
	Acute PAF ^e^	1.00	1.00	/	/	0.70	/	/	This study
Fe	N ^a^	14	229	/	70	/	19	/	[50,51,52,56,57,58,59,60,61,62,63,64,71,72]
	Range ^b^	0.004–1.59	0.013–78.42	/	0.12–3.61	/	0.18–3.68	/	
	GM ^c^	0.11	7.74	/	0.72	/	1.48	/	
	SD ^d^	0.083	14.67	/	0.35	/	0.79	/	
	Acute PAF ^e^	0.82	0.98	/	0.93	/	0.95	/	This study
	Chronic PAF ^f^	0.82	1.00	/	0.94	/	0.97	/	
Mn	N ^a^	17	224	/	70	12	22	34	[49,50,51,52,53,54,57,58,59,60,61,62,64,65,66,71,72,73,74]
	Range ^b^	0.004–0.09	0.010–2.72	/	0.04–0.20	0.01–2.5	0.0003–0.5	0.071–0.23	
	GM ^c^	0.034	0.32	/	0.07	1.44	0.022	0.16	
	SD ^d^	0.08	0.25	/	0.034	0.72	0.009	10.54	
	Acute PAF ^e^	0.48	0.85	/	0.64	0.93	0.37	0.78	This study
Zn	N ^a^	88	255	4	82	15	22	34	[28,48,49,50,51,52,53,54,55,56,57,58,59,60,61,62,63,64,65,66,67,68,69]
	Range ^b^	0.01–1.87	0.005–1.35	0.007–0.110	0.02–0.12	0.01–0.032	0.01–0.12	0.0072–0.097	
	GM ^c^	0.39	0.13	0.039	0.041	0.015	0.047	0.037	
	SD ^d^	0.545	0.078	0.02	0.009	0.005	0.008	25.25	
	Acute PAF ^e^	0.98	0.90	0.73	0.74	0.57	0.76	0.72	This study

Note: ^a^ Numbers of samples collected in this study; ^b^ range of concentrations of samples collected in this study; ^c^ mean concentrations of all samples collected in this study; ^d^ standard deviation of concentrations of all samples collected in this study; ^e^ acute PAF value calculated by use of mean concentrations of all samples collected in this study; ^f^ chronic PAF value calculated by use of mean concentrations of all samples collected in this study.

## Data Availability

The data presented in this study are available in article and Supplementary Materials.

## Supplementary Materials

The following are available online at https://www.mdpi.com/article/10.3390/ijerph192316125/s1, Table S1. Acute toxicity data information for the studied transition metals in South Asia (unit: mg/L), Table S2. Chronic toxicity data information for the studied transition metals in South Asia (unit: mg/L), Figure S1. (a) Acute HC5 values derived based on NPKDE-SSDs for 5% of the species for Cd (●), Cu (●), Hg(●), Mn(●), Fe (●), and Zn (●); (b) chronic HC5 values derived based on NPKDE-SSDs for 5% of the species for Cd (●), Cu (●), and Fe (●).
