# Supplementary material for: Water Quality Criteria and Ecological Risk Assessment of Typical Transition Metals in South Asia
Source: Int J Environ Res Public Health. 2022 Dec 2;19(23):16125. doi: 10.3390/ijerph192316125 (PMC9739192; doi:10.3390/ijerph192316125)
Supplement: Supplementary file 1 [file ijerph-19-16125-s001.zip › ijerph-2009616-supplementary.pdf]

Supporting Information

Water Quality Criteria and Ecological Risk Assessment of Typical Transition Metals in South Asia

Ying Wang [1], Tanjena Rume [1], S. M. Didar-Ul Islam [2], Wenhong Fan [1,3], Jiangyue Wu [4,*] and Xiaomin Li [1,*]

Authors affiliations:

[1] School of Space and Environment, Beihang University, Beijing 100191, China
[2] School of Civil Engineering and Geosciences, Newcastle University, Newcastle upon Tyne NE1 7RU, UK
[3] Beijing Advanced Innovation Center for Big Data-Based Precision Medicine, Beihang University, Beijing 100191, China
[4] National Marine Hazard Mitigation Service, Ministry of Natural Resource of the People's Republic of China, Beijing 100194, China

Corresponding author:

Jiangyue Wu, E-mail: wujiangyue2005@126.com;

Xiaomin Li, E-mail: xiaominli@buaa.edu.cn.

Table S1. Acute toxicity data information for the studied transition metals in South Asia (unit: mg/L).

Metals	Parameters	All species	Plants	Fish	Molluscs	Amphibians	Crustaceans	Insects	Invertebrates	Worms
Cd	N[a]	76	3	28	9	4	14	4	8	5
	Min[b]	0.0027	0.015	0.84	0.0027	2.23	0.02	3.12	0.07	3.09
	Max[c]	300	3.64	300	10	8.6	2.08	26.63	1.4	100
	μ[d]	12.14	1.53	22.57	4.18	4.95	0.41	16.46	0.64	30.99
	σ[e]	1354.82	3.57	3162.57	14.76	10.83	0.46	144.70	0.22	1610.32
Cu	N[a]	66	3	33	3	5	12	3	6	1
	Min[b]	0.0056	0.99	0.03	0.15	0.059	0.0056	0.17	0.049	-
	Max[c]	900.78	1.025	900.78	5	5.47	25.93	8.67	443.31	-
	μ[d]	23.005	0.85	30.66	2.84	1.68	2.24	5.23	74.11	-
	σ[e]	15041.05	0.082	24505.83	6.10	5.09	55.65	19.98	327175.75	-
Hg	N[a]	59	5	24	8	5	9	4	1	2
	Min[b]	0.0016	0.028	0.045	0.0016	0.13	0.0024	1.4	-	0.12
	Max[c]	17.97	3.80	6.28	4.42	17.97	0.71	7.61	-	1.41
	μ[d]	1.28	0.82	1.011	1.14	3.97	0.16	3.48	-	0.77
	σ[e]	7.20	0.011	1.98	2.63	61.32	0.057	8.009	-	0.83
Mn	N[a]	12	4	2	1	2	3	-	-	1
	Min[b]	2.62	2.62	420.14	-	15.78	9.29	-	-	-
	Max[c]	2988.36	100	2988.36	-	63.28	40.89	-	-	-
	μ[d]	315.87	36.91	1704.25	-	39.53	24.29	-	-	-
	σ[e]	721269.9	1905.48	3297874	-	1127.85	251.68	-	-	-
Fe	N[a]	13	4	1	2	3	2	-	-	-
	Min[b]	0.21	2.40	-	0.56	0.61	6.66	-	-	-
	Max[c]	1950	1950	-	17.94	79.67	13.66	-	-	-
	μ[d]	167.94	510.58	-	9.25	33.75	10.16	-	-	-
	σ[e]	287435	921915.60	-	150.98	1685.19	24.54	-	-	-
Zn	N[a]	55	3	26	9	4	6	1	5	1
	Min[b]	0.0069	0.55	0.008	0.39	0.0069	0.17	-	0.68	-
	Max[c]	267.25	21.25	260	267.25	23.66	1.75	-	14	-
	μ[d]	34.28	8.35	42.47	58.89	11.64	0.69	-	8.46	-
	σ[e]	3201.82	126.63	3086.32	8192.24	123.22	0.37	-	30.75	-

Note: [a]Number of species included in model, [b]Minimum value (mg/L) of species toxicity data included in model for the metal, [c]Maximum value(mg/L) of species toxicity data included in model for the metal, [d]Mean value (mg/L)of species toxicity data included in model for the metal, [e]Standard variance (mg/L)of species toxicity data included in model for the metal.

Table S2. Chronic toxicity data information for the studied transition metals in South Asia (unit: mg/L).

Metals	Parameters	All species	Plants	Fish	Molluscs	Crustaceans	Insects	Invertebrates	Worms	Amphibians
Cd	N[a]	34	7	13	2	5	3	2	2	-
	Min[b]	0.00082	0.001	0.003	0.23	0.00082	0.003	0.0199	0.25	-
	Max[c]	538	0.33	538	0.23	0.00613	0.43	0.67	11.20	-
	μ[d]	16.92	0.081	43.21	0.23	0.0034	0.15	0.343	5.72	-
	σ[e]	8492.08	0.014	22131.35	0	3.91	0.061	0.21	60.022	-
Cu	N[a]	38	8	12	7	5	2	2	2	-
	Min[b]	0.0084	0.0089	0.022	0.013	0.0086	0.016	0.0084	0.030	-
	Max[c]	37.52	0.612	37.52	0.13	0.059	0.058	3.82	4.22	-
	μ[d]	1.27	0.106	3.22	0.079	0.033	0.037	1.92	2.13	-
	σ[e]	37.28	0.043	116.71	0.0031	0.00052	0.00087	7.26	8.77	-
Fe	N[a]	16	3	5	-	3	2	-	1	2
	Min[b]	0.0016	0.01	0.0016	-	0.96	89.85	-	10	7.79
	Max[c]	560	16.94	560	-	30	282.85	-	10	10.045
	μ[d]	66.71	7.07	122.66	-	10.79	186.34	-	10	8.92
	σ[e]	22278.36	77.56	60154.099	-	276.81	18624.02	-	0	2.54

Note: [a]Number of species included in model, [b]Minimum value (mg/L) of species toxicity data included in model for the metal, [c]Maximum value (mg/L) of species toxicity data included in model for the metal, [d]Mean value (mg/L) of species toxicity data included in model for the metal, [e]Standard variance of species toxicity data included in model for the metal.

(a)

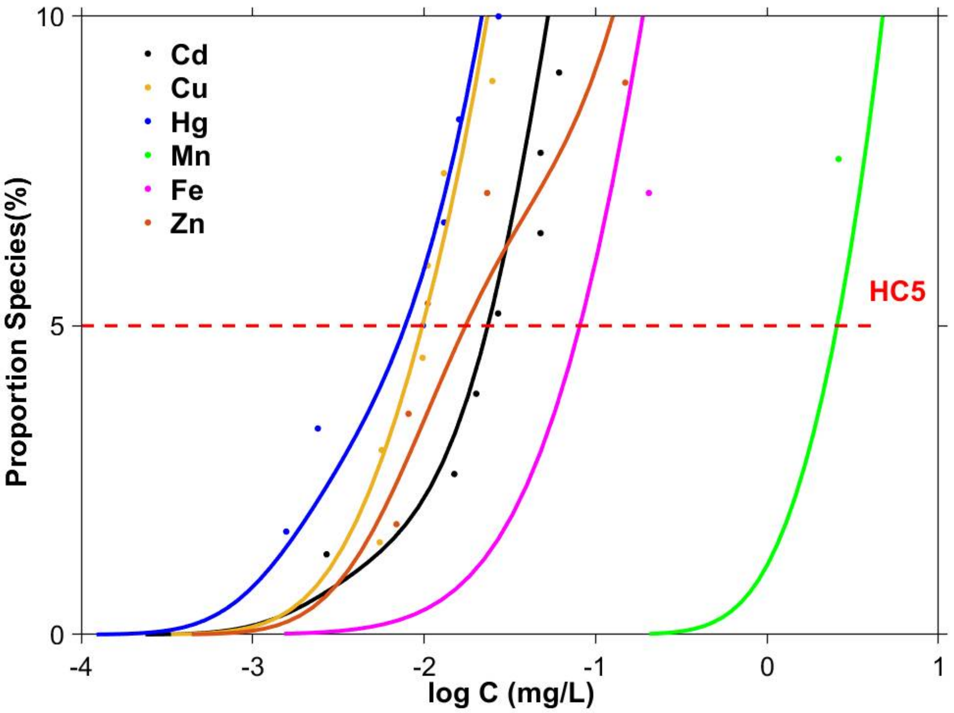

(b)

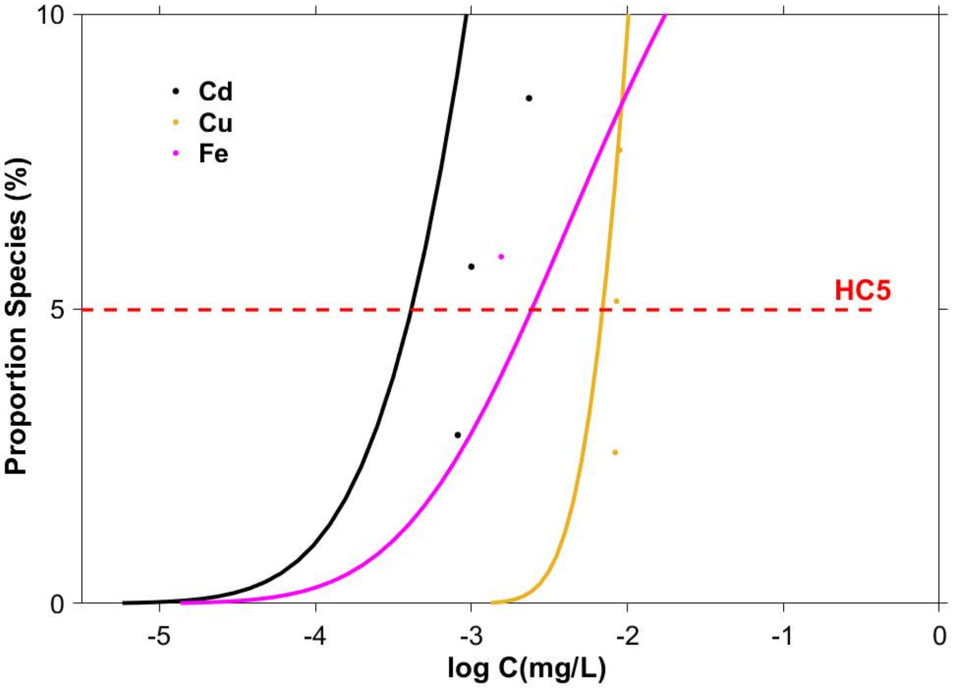

Figure S1. (**a**) Acute HC5 values derived based on NPKDE-SSDs for 5% of the species for Cd (●), Cu (●), Hg (●), Mn(●), Fe (●) and Zn (●); (**b**) chronic HC5 values derived based on NPKDE-SSDs for 5% of the species for Cd (●), Cu (●) and Fe (●).
